# *In silico* and *in vitro* chemometrics, cell toxicity and permeability of naringenin 8-sulphonate and derivatives

**DOI:** 10.3389/fphar.2024.1398389

**Published:** 2024-07-24

**Authors:** Tiago Macedo, Fátima Paiva-Martins, Patrícia Valentão, David M. Pereira

**Affiliations:** ^1^ REQUIMTE/LAQV, Laboratório de Farmacognosia, Departamento de Química, Faculdade de Farmácia, Universidade do Porto, Porto, Portugal; ^2^ REQUIMTE/LAQV, Departamento de Química e Bioquímica, Faculdade de Ciências, Universidade do Porto, Porto, Portugal

**Keywords:** *Parinari excelsa*, naringenin 8-sulphonate, permeability, pharmacokinetics, toxicity

## Abstract

**Background:**

Sulphur containing natural compounds are among the most biologically relevant metabolites *in vivo*. Naringenin 8-sulphonate from *Parinari excelsa* Sabine was evaluated in a previous work, demonstrating ability to act as a natural anti-inflammatory. Although the interference of this molecule against different inflammatory mediators was described, there is no information regarding its potential toxicity and pharmacokinetics, which are essential for its capacity to reach its therapeutic targets. In fact, despite the existence of reports on naringenin ADMET properties, the influence of sulphation patterns on them remains unknown.

**Objectives:**

This work aims to assess the *in vitro* pharmacokinetic and toxicological behavior of naringenin 8-sulphonate, as well as to understand the importance of the presence and position of the sulphur containing group for that.

**Methods:**

Naringenin 8-sulphonate physicochemical and ADMET properties were investigated using *in silico* tools and cell-based *in vitro* models. At the same time, naringenin and naringenin 4’-*O*-sulphate were investigated to evaluate the impact of the sulphonate group on the results. ADMETlab 2.0 *in silico* tool was used to predict the compounds’ physicochemical descriptors. Pharmacokinetic properties were determined experimentally *in vitro*. While MRC-5 lung fibroblasts and HaCaT keratinocytes were used to evaluate the cytotoxicity of samples through MTT and LDH assays, Caco-2 human intestinal epithelial cells were used for the determination of genotoxicity, through alkaline comet assay, and as a permeability model to assess the ability of compounds to cross biological barriers.

**Results:**

Experimental determinations showed that none of the compounds was cytotoxic. In terms of genotoxicity, naringenin 8-sulphonate and naringenin caused significant DNA fragmentation, whereas naringenin 4’-*O*-sulphate did not. When it comes to permeability, the two sulphur-containing compounds with a sulphur containing group were clearly less capable to cross the Caco-2 cell barrier than naringenin.

**Conclusion:**

In this study, we conclude that the sulphur containing group from naringenin 8-sulphonate is disadvantageous for the molecule in terms of ADMET properties, being particularly impactful in the permeability in intestinal barrier models. Thus, this work provides important insights regarding the role of flavonoids sulphation and sulphonation upon pharmacokinetics and toxicity.

## 1 Introduction

Natural products have been of major importance in drug discovery, with many secondary metabolites having several biological properties reported. However, some natural compounds are capable of causing harmful effects in the human organism (Madariaga-Mazón et al., 2019). An efficient strategy to minimize the risk of those undesirable effects is to avoid the administration of chemically-complex natural extracts, which contain often inactive and potentially toxic compounds in their composition. For this purpose, the isolation of the compound of interest arises as a good strategy (Rasoanaivo et al., 2011). Nevertheless, it is important to ensure that the bioactive compounds will not lead to toxic effects themselves, through the study of their toxicological profile. Thus, as it happens with synthetic compounds, the potential toxicity of natural molecules must always be assessed before their application in therapeutics (Madariaga-Mazón et al., 2019).

In fact, concerning compounds safety and efficacy, several aspects should be considered besides biological activity, including absorption, distribution, metabolism, excretion and toxicity (ADMET) (Li et al., 2019; Gaohua et al., 2021; Zhong, 2017; Dulsat et al., 2023). This study is further warranted if we consider that 90% of the drug failures along the past decade were due to a poor pharmacokinetic profile (Dulsat et al., 2023). To avoid drugs failure during late stages of development, and the associated economical losses, ADMET prediction tools have become as an essential part of drug research (Dulsat et al., 2023).

The evergreen tree species *P. excelsa* Sabine is distributed across several African countries, its bark being widely used in folk medicine for the treatment of many health conditions, including anemia, diabetes, malaria, heart problems and stomach ache (Kamuhabwa et al., 2000; Ndiaye et al., 2008; Feitosa et al., 2012; Hwang et al., 2020). As previously reported by our group (Macedo et al., 2023), naringenin 8-sulphonate isolated from the stem bark of *P. excelsa* scored positively in a set of experiments to assess its anti-inflammatory potential.

*P. excelsa* belongs to a growing group of plants with ethnopharmacological relevance that have sulphur containing flavonoids in their phytochemical composition. Other examples are: *Polygonum hydropiper* L., a plant used for the treatment of many health conditions, such as anxiety, gynaecological disorders, pain and ulcers (Bairagi et al., 2022), where isorhamnetin 3-*O*-sulphate may be found (Teles et al., 2018); *Wissadula periplocifolia* (L.), used in folk medicine for the treatment of liver diseases (Hossain et al., 2023), with several flavonoid sulphates reported in its composition, such as acacetin 7-*O*-sulphate, hypoaletin 3′-*O*-methyl ether 8-*O*-sulphate and isoscutellarein 8-*O*-sulphate (Teles et al., 2018); *Bixa Orellana* L., a plant used as analgesic, hemostatic and wound healing (Coelho Dos Santos et al., 2022), which contains apigenin 7-*O*-sulphate and hypoaletin 8-*O*-sulphate (Teles et al., 2018).

Despite the promising anti-inflammatory activity revealed by naringenin 8-sulphonate from *P. excelsa* (Macedo et al., 2023), no information could be found in literature about the toxicity and pharmacokinetic behavior of this compound. For this reason, this work is focused on the prediction of ADMET properties, evaluation of the cytotoxicity in non-cancerous cell lines, genotoxicity and permeability across a biological barrier. Furthermore, the same experimental design was followed for the evaluation of naringenin and naringenin 4′-*O*-sulphate, thus contributing to understand the impact of the presence and position of the sulphur group.

## 2 Materials and methods

### 2.1 General procedures, materials and chemicals

Agarose, low gelling temperature agarose, 3-(4,5-dimethylthiazol-2-yl)-2,5-diphenyltetrazolium bromide (MTT), methylmethanesulphonate (MMS), β-Nicotinamide adenine dinucleotide - reduced disodium salt hydrate (NADH), dimethyl sulphoxide (DMSO), 2-(4-amidinophenyl)-6-indolecarbamidine dihydrochloride (DAPI), isopropanol, lucifer yellow CH di-potassium salt, naringenin, sodium pyruvate, Triton X-100 and trypan blue were purchased from Sigma-Aldrich (St Louis, MO, United States). Dulbecco’s Modified Eagle Medium (DMEM) high glucose GlutaMAX™ supplemented with pyruvate, Minimum Esssential Medium (MEM) GlutaMAX™ Supplement, FBS, penicillin-streptomycin solution (penicillin 5,000 units mL^-1^ and streptomycin 5,000 μg mL^-1^) and trypsin-EDTA (0.25%) - phenol red were purchased from GIBCO, Invitrogen™ (Grand Island, NY, United States). Naringenin 8-sulfonate was isolated from *P. excelsa* through preparative thin layer chromatography (TLC) eluted with isobutanol/water/acetic acid (6:2:1), as described in Macedo et al. (2023). Naringenin 4′-*O*-sulphate was synthesized following the protocol described previously by the group in Macedo et al. (2023). Purity of naringenin 4′-*O*-sulphate and naringenin 8-sulphonate was confirmed by HPLC, being superior to 90% in both cases.

### 2.2 Cell culture and seeding

MRC-5 human lung fibroblast cells (Sigma-Aldrich, St. Louis, MO, United States) were cultured in MEM, while HaCaT human keratinocyte cells (ATCC, Manassas, VA, United States) and Caco-2 human intestinal epithelial cells (American Type Culture Collection, LGC Standards S.L.U., Spain) were cultured in DMEM. Both medium were supplemented with 10% of FBS and 1% of penicillin/streptomycin. Cells were incubated in 75 cm^2^ cell culture flasks, which were placed in an incubator at 37°C, with a humidified atmosphere of 5% CO_2_. For the experiments, cells were seeded for 24 h in 96-well plates. MRC-5 and Caco-2 were seeded at 20,000 cells/well and HaCaT were seeded at 15,000 cells/well.

### 2.3 Cell viability assay

Toxicity of compounds, at 50 and 100 μM, was assessed against non-cancerous cell lines through the MTT assay after 24 h of incubation, following the protocol described in Macedo et al., (2023), with slight modifications. Thus, cells were exposed to MTT for 2 h, in the case of MRC-5, and 1 h 30 min, in the case of HaCaT. This assay was also performed to evaluate the response of Caco-2 cells to compounds at concentrations from 125 to 1,000 μM during the permeability assay, these cells being incubated for 4 h with the samples and exposed to MTT for 2 h. Results were expressed as percentage of viability compared to the control and correspond to the mean of, at least, three independent experiments performed in triplicate.

### 2.4 Membrane integrity assay

Membrane integrity was evaluated based on the LDH release to the extracellular medium. Sodium pyruvate solution was added to 50 μL of supernatant collected during the viability assay protocol. Then, NADH was added and the absorbance at 340 nm was monitored for 3 min to evaluate the conversion of pyruvate to lactate (Silva et al., 2017). Triton X-100 1% was incubated for 30 min to be used as positive control for cell lysis. Results were expressed as fold-increase compared to negative control and reflect the mean of three independent experiments, each performed in triplicate.

### 2.5 DNA damage assay

Caco-2 cells were incubated for 24 h in 12 well plates at a density of 350,000 cells/well. After this, they were incubated with each sample and negative control for 24 h and collected using 500 μL of trypsin (0.25%)-ethylenediaminetetraacetic acid (EDTA) (0.038%), which was then inactivated with 1 mL of Hanks’ Balanced Salt Solution (HBSS). The cell suspension was centrifuged at 1,200 rpm during 8 min at room temperature, supernatant was discarded, and cells were resuspended in 100 μL of HBSS.

Electrophoresis was carried out to evaluate the extent of DNA fragmentation. For this, agarose-coated slides were prepared using an 1% agarose solution. A volume of 20 μL from the cell suspension was involved in 170 μL of 1% low gelling temperature agarose solution and applied to the slides, which were then placed at 4°C until solidification. Slides were submerged in a lysis solution (2.5 M NaCl, 0.1 M EDTA, 10 mM Tris-HCl, pH 10) with 1% Triton-X 100, at 4°C and sheltered from the light, for 105 min. After this, slides were submerged in the electrophoresis buffer (300 mM NaOH, 1 mM EDTA, pH 13) for 40 min. A Sub-Cell GT Horizontal Electrophoresis System 15 × 15 cm tray (Bio-Rad Laboratories Lda, Oeiras, Portugal) was used to run the electrophoresis at 25 V and 240 mA during 30 min in the dark. In the end of the electrophoresis, slides were submerged for three times in a neutralization solution (0.4 M Tris-HCl, pH 7.5), during 5 min each.

For the fluorescence microscopy analysis, DAPI was added for 15 min at 0.5 μg mL^-1^, the slides being washed once with HBSS in the end. Images were acquired using a Nikon Eclipse Ts2R-FL inverted microscope with a Retiga R1 camera (01-RET-R1-RM) and CFI Plan Fluor ×10 DIC objective. DNA damage was calculated using the AutoComet algorithm (Barbe et al., 2023) by dividing the amount of fluorescence signal correspondent to the DNA content in the comet’s tail by the signal correspondent to the total DNA in the comet. MMS 500 μM, incubated for 30 min, was used as positive control for DNA fragmentation and consequent DNA tail formation.

### 2.6 Permeability assay

Caco-2 cells were seeded for 21 days at 112,000 cells/well in Corning^®^ 3460 Transwell^®^ 12 mm Polyester Membrane Inserts (Corning Incorporated, ME, United States). At every other day, culture medium was replaced and the transepithelial electrical resistance was measured to ensure values above 400 ohms until the day of the assay, to guarantee membrane integrity. For quality control purposes, Lucifer Yellow was used at 100 μg mL^-1^ in the day of the assay. Briefly, one of the inserts was incubated with Lucifer Yellow during 1 h and, after that, its concentration was measured in the basolateral chamber, based on fluorescence intensity (λexc = 485 nm and λem = 535 nm). Lucifer Yellow passage, from the apical to the basolateral chamber equal or lower than 3% was considered indicative of a well-defined cell barrier phenotypically translated as good membrane integrity.

Treatments were applied in the transwells apical chamber and the basolateral chamber was filled with HBSS with calcium (1.26 mM) and magnesium (0.81 mM). At specific timepoints, namely 10, 20, 30, 60, 90, 120 and 180 min, half of the volume from the basolateral chamber was collected and replaced by the same volume of HBSS. In the last time point, at 240 min, content of both apical and basolateral compartments was collected to calculate the final mass balance. Transwells were maintained in agitation at 50 rpm, 37°C and 5% CO_2_ between the timepoints.

Concentration of each analyte in the different compartments was determined by HPLC-DAD using a Gilson Medical Electronics HPLC system (Villiers le Bel, France), with a Spherisorb ODS^®^ (250 × 4.6 mm, 5 μm particle size, 100 Å pore size) column (PSS831915, Waters, Dublin, Ireland). Elution was performed for 7 min, with a flow rate of 900 μL min^-1^. A mixture of methanol: water-formic acid (1%) was used as mobile phase, in a proportion of 65:35 for naringenin and 47:53 for naringenin 4’-*O*-sulphate and naringenin 8-sulphonate. Quantifications were performed at 285 nm.

Results were obtained from three independent experiments and each sample was analysed in triplicate. Apparent permeability (*P*_*app*_) coefficient (cm s^-1^) was calculated based on the equation *P*_*app*_ = ΔQ/Δt×(1/(A×C_0_)), where ΔQ/Δt represents the amount of compound over time (mol s^-1^), A is the monolayer area (cm^2^) and C0 (mol mL^-1^) is the initial drug concentration of the apical side (Nait et al., 2009; Silva et al., 2020).

### 2.7 *In silico* models and data analysis

Python 3.9 was used to extract from COCONUT database (https://coconut.naturalproducts.net/) the available data concerning sulphur containing flavonoids. First, all the molecules belonging to the phenylpropanoids were selected, which yielded 56,527 molecules. From here, they were further narrowed down to flavonoids, yielding 27,690 compounds. Finally, filtering focusing on molecules bearing sulphur resulted in 585 molecules being extracted, which were divided in several subclasses, including isoflavonoids, aurones, neoflavonoids, among others. From those, only the subclasse “Flavonoids” (flavonols, flavones, flavanones, among others) was kept, to which the synthetic molecules used in this study were added. The different physicochemical and topographical features were generated using the Python RdKit library (version 2022.09.5) (Landrum, 2010), using each molecule’s smile notation as input. Data was cleaned using an in-house cleaning function that involves dropping absent/null cells and use of the SimpleImputer (strategy = “median”) and StandardScaler methods from the Sci-kit learn library (Pedregosa et al., 2011). The resulting database is provided as Supplementary Material S1. Dimensionality reduction was done using a t-distributed stochastic neighbor embedding (t-SNE) performed using sci-kit learn with standard parameters, n_components = 2 and n_jobs = −2.

The ADMETlab 2.0 online prediction tool (Xiong et al., 2021) was used to estimate compounds ADMET properties. ImageJ 1.53t and AutoComet (Barbe et al., 2023) were used for immunofluorescence image processing. Violin plot graphical design was performed using Numpy (Harris et al., 2020), Pandas (McKinney and WaJ, 2010), Seaborn (Waskom, 2021) and Matplotlib (Hunter, 2007) Python libraries. The remaining graphs were designed using Graph Pad Prism 8.4.2 (San Diego, CA, United States ). Analysis was performed using data from, at least, three independent experiments. Data normality was confirmed using Shapiro-Wilk normality test and comparison between treatments was performed using ANOVA test. Positive controls were compared to negative controls through the *t*-test. Grubb’s test was used for outliers’ detection.

## 3 Results and discussion

### 3.1 Chemometrics

Sulphur containing natural compounds are widely recognized for their importance in physiological processes occurring in several species, including humans (Francioso et al., 2020). Since during our previous work naringenin 4’-*O*-sulphate and naringenin 8-sulphonate exhibited uncommon properties when compared to other sulphur containing flavonoids (Macedo et al., 2023), in this work the chemical space of these molecules was studied to understand if they could be highlighted from the others based on their physicochemical features.

**FIGURE 1:**
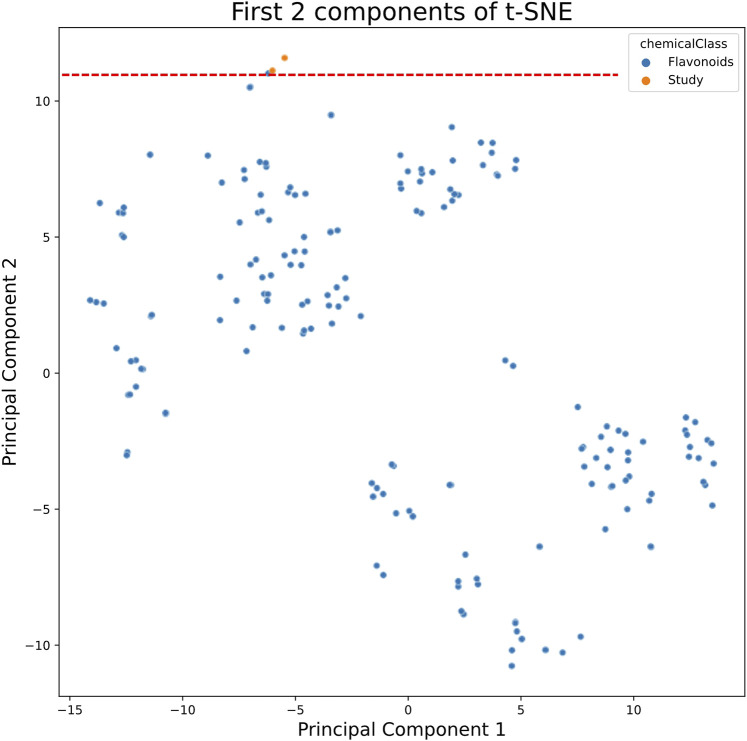
t-SNE algorithm applied to sulphur containing flavonoids from COCONUT database (presented in blue) and the molecules under study naringenin 4’-*O*-sulphate and naringenin 8-sulphonate (presented in orange).

Thus, a t-SNE algorithm was applied to naringenin 8-sulphonate, naringenin 4’-*O*-sulphate and other 185 sulphur containing flavonoids (Supplementary Material S1), selected as described in the “Materials and methods” section. Indeed, among the 185 sulphur-containing flavonoids that we were able to compile from the available literature, the two compounds under study are placed in a group of four outliers in this chemical space, as can be observed in Figure 1. The other two outliers are hesperetin 3’-*O*-sulphate and [6,7-dimethoxy-2-(4-methoxyphenyl)-4-oxo-3,4-dihydro-2H-1-benzopyran-5-yl]oxidanesulphonic acid, which are also sulphur containing flavanones, the first being particularly similar to the compounds of this study. These data reinforce the interest to study the physicochemical features and pharmacodynamic properties of naringenin sulphur containing derivatives.

### 3.2 Physicochemical descriptors and drug-likeness prediction

Physicochemical descriptors and ADMET properties of naringenin 8-sulphonate from *P. excelsa* were assessed. Additionally, naringenin and naringenin 4’-*O*-sulphate were studied to compare the results and understand the importance of the presence and position of the sulphur group.

**FIGURE 2:**
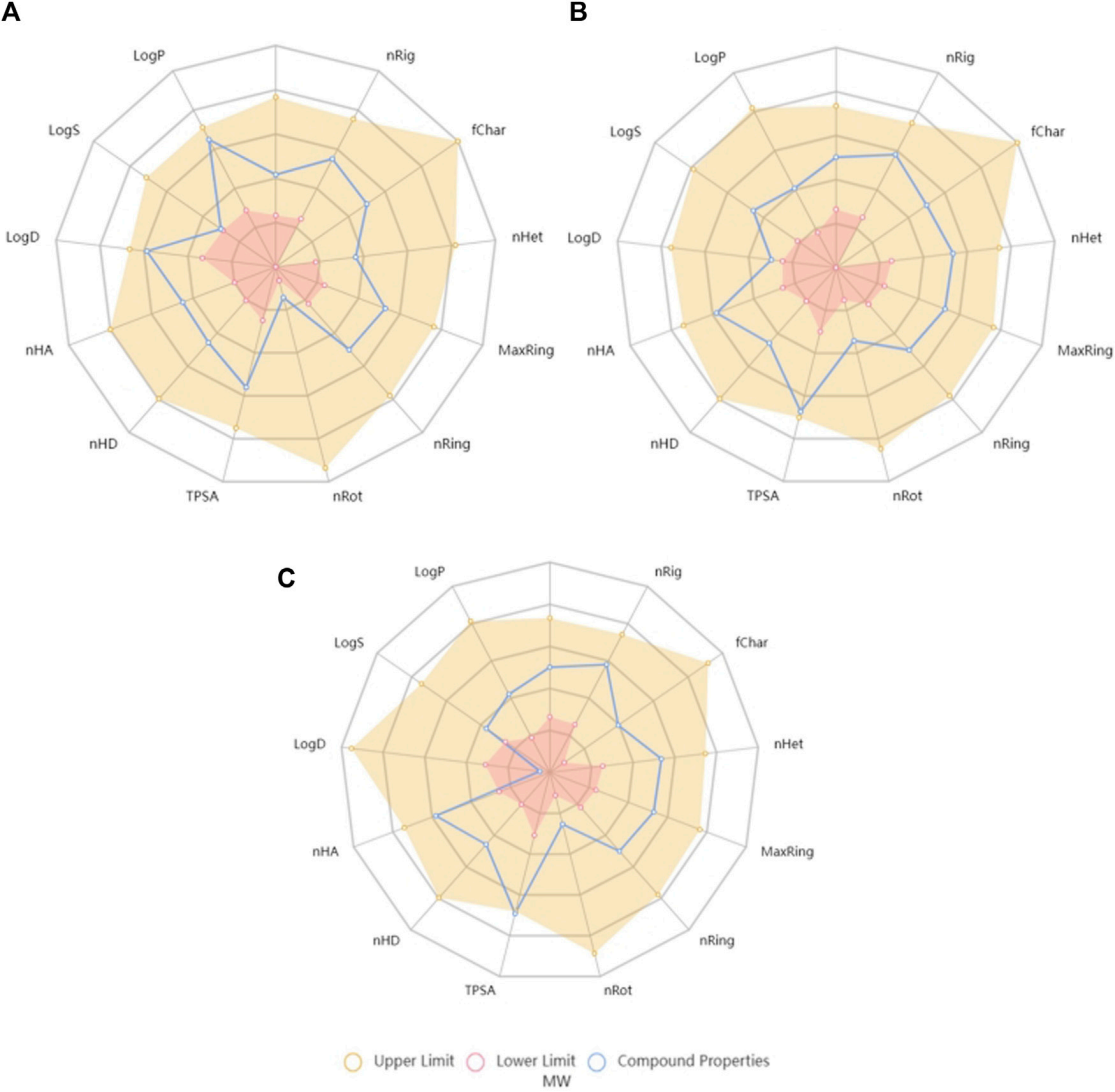
Radar charts generated by ADMETlab 2.0 tool with the chemical space of naringenin **(A)**, naringenin 4’-*O*-sulphate **(B)** and naringenin 8-sulphonate **(C)** on the lower and upper limits of crucial features for the drug-likeness. MW, molecular weight; nRig, number of rigid bonds; fChar, Formal Charge; nHet, number of heteroatoms; MaxRing, number of atoms in the larger ring; nRing, number of rings; nRot, number of rotable bonds; TPSA, topological polar surface area; nHD, number of hydrogen bond donors; nHA, number of hydrogen bond acceptors; logD, logarithm of distribution coefficient; logS, logarithm of aqueous solubility value; logP, logarithm of partition coefficient.

Currently, there are several tools available for the prediction of ADMET parameters (Dulsat et al., 2023). One of these tools is ADMETlab 2.0, which may be highlighted from many others due to the precision, accuracy and wide coverage of parameters (Dulsat et al., 2023). Several physicochemical properties are considered by this tool during the evaluation of molecules and it schematizes the interval of values that each molecule should fulfil regarding several features, such as molecular weight (MW), partition coefficient (logP) or topological polar surface area (TPSA), which are parameters that have been considered along drug discovery processes of world-famous pharmaceutical industries, originating drug-likeness rules, such as Pfizer rule and GSK rule, which are also verified by ADMETlab 2.0 (Xiong et al., 2021). As may be seen in Figure 2, naringenin and naringenin 4’-*O*-sulphate are in the acceptable range of values for all the parameters, while naringenin 8-sulphonate has distribution coefficient (logD) and TPSA values that are outside the optimal range for a drug.

In order to transform the drug-likeness concept in a numerical score, and consequently quantify and compare values, ADMETlab 2.0 calculates the quantitative estimate of drug-likeness (QED), whose formula is based on many drug-likeness related properties, including MW, logP and TPSA. According to the tool, attractive compounds should have a QED value superior to 0.67. Regarding the tested compounds, obtained values were 0.742, 0.715, and 0.689 for naringenin, naringenin 4’-*O*-sulphate and naringenin 8-sulphonate, respectively, all of them marginally exceeding the desirable threshold.

Besides the previously mentioned traits, among the large amount of other parameters predicted by the ADMETlab 2.0, two of them were especially relevant in this work, namely Ames toxicity, which indicates the mutagenic potential of a molecule (Hebert et al., 2015), and Caco-2 permeability, which was experimentally determined herein. Regarding Ames toxicity, ADMETlab 2.0 expresses the probability of a compound being toxic and qualifies it as excellent (meaning unlikely toxicity) when it is less than 0.3, medium when it is between 0.3 and 0.7, and poor when it is above 0.7. Naringenin 8-sulphonate probability was defined as 0.079, fitting in the best category. This was also the situation of naringenin 4′-*O*-sulphate, whose probability was even lower (0.011). On the other hand, naringenin mutagenicity probability was 0.342, which classifies the compound as medium in this parameter. MMS, which was used in our experimental work as mutagenic positive control, had a mutagenicity probability of 0.968. When it comes to Caco-2 permeability, the tool exhibits the values in log cm s^-1^ units and considers that values above −5.15 translates as adequate permeability. Thus, naringenin 8-sulphonate, with a predicted permeability value of −5.432, and its isomer naringenin 4’-*O*-sulphate, with −5.431, may be considered as poor. In opposition, the score of −4.803 obtained by naringenin is a good indicator for its ability to cross biological barriers.

### 3.3 Cell viability assay

**FIGURE 3:**
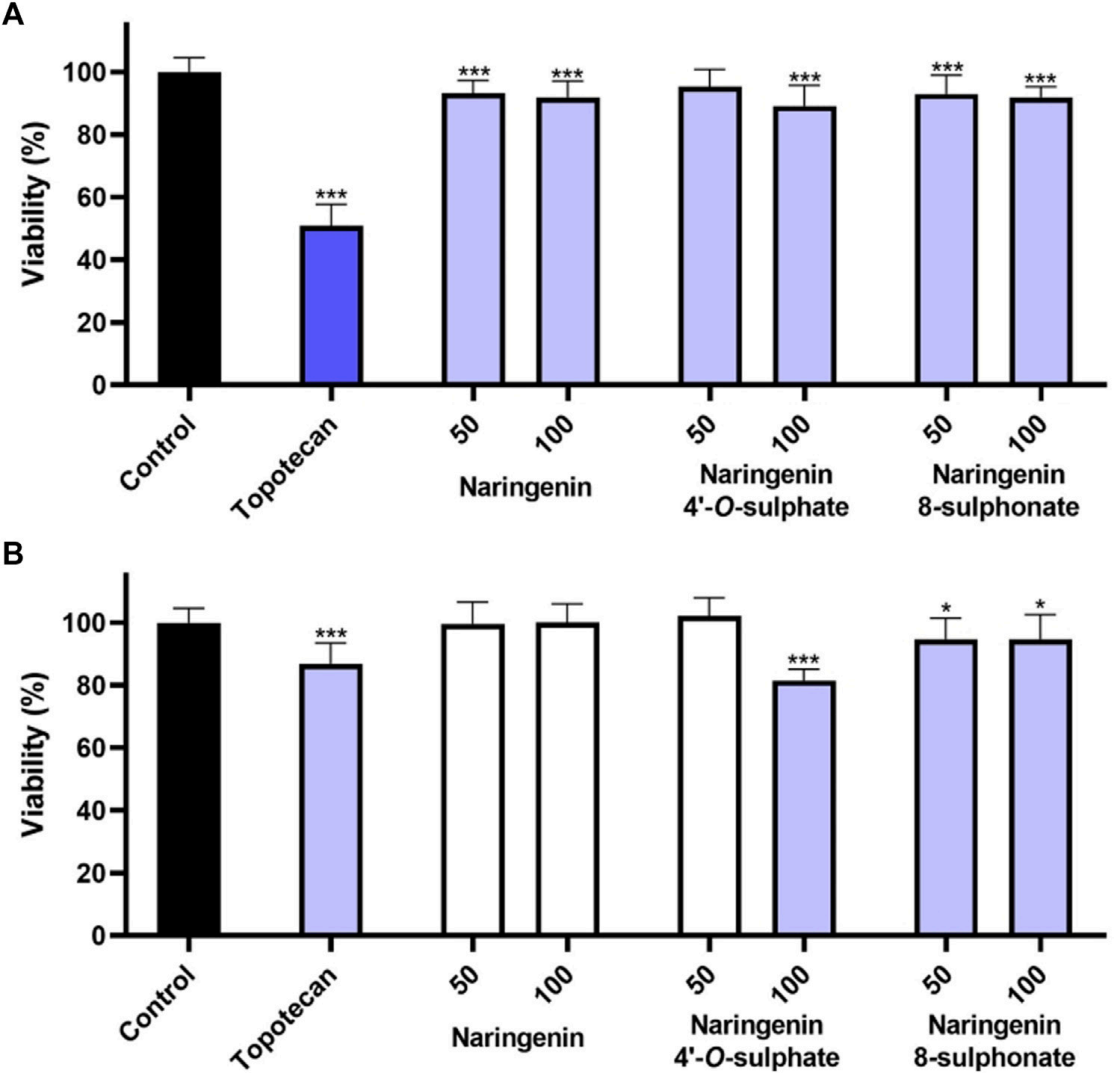
Effects of naringenin, naringenin 4’-*O*-sulphate and naringenin 8-sulphonate, at concentrations of 50 and 100 μM, on the viability of HaCaT **(A)** and MRC-5 **(B)** cells. Topotecan (100 μM) was used as positive control. Results are expressed as mean of, at least, three independent experiments, each performed in triplicate. *p < 0.05, ***p < 0.001.

The impact of compounds on non-cancer cell lines HaCaT and MRC-5 was assessed through the MTT assay. As skin keratinocytes and lung fibroblasts, respectively, these cell lines come from two organs that are often a path for the application of anti-inflammatory drugs. In HaCaT, the decrease of cell viability was less than 15% for all the tested conditions. While naringenin 8-sulphonate decreased cell viability to 92.99% and 91.95% at 50 and 100 μM, respectively, the values obtained with naringenin were 93.20% and 91.92% and with naringenin 4’-*O*-sulphate were 95.42% and 89.20%. These results indicate that the three compounds have a very similar impact on HaCaT, which seems not to depend neither on the presence, nor on the position of the sulphur containing group (Figure 3A). Regarding the effects on MRC-5, naringenin 8-sulphonate caused, once again, a slight decrease on cell viability, reducing the values to 94.77% and 94.71% at 50 and 100 μM, respectively, which are acceptable. In this case, naringenin 4’-*O*-sulphate caused a more pronounced viability decrease, reaching 81.48% at 100 μM, still being within the range of acceptable loss of viability (ISO 10993-5, 2009). Naringenin did not elicit any significant decrease on MRC-5 cell viability (Figure 3B).

### 3.4 Membrane integrity

**FIGURE 4:**
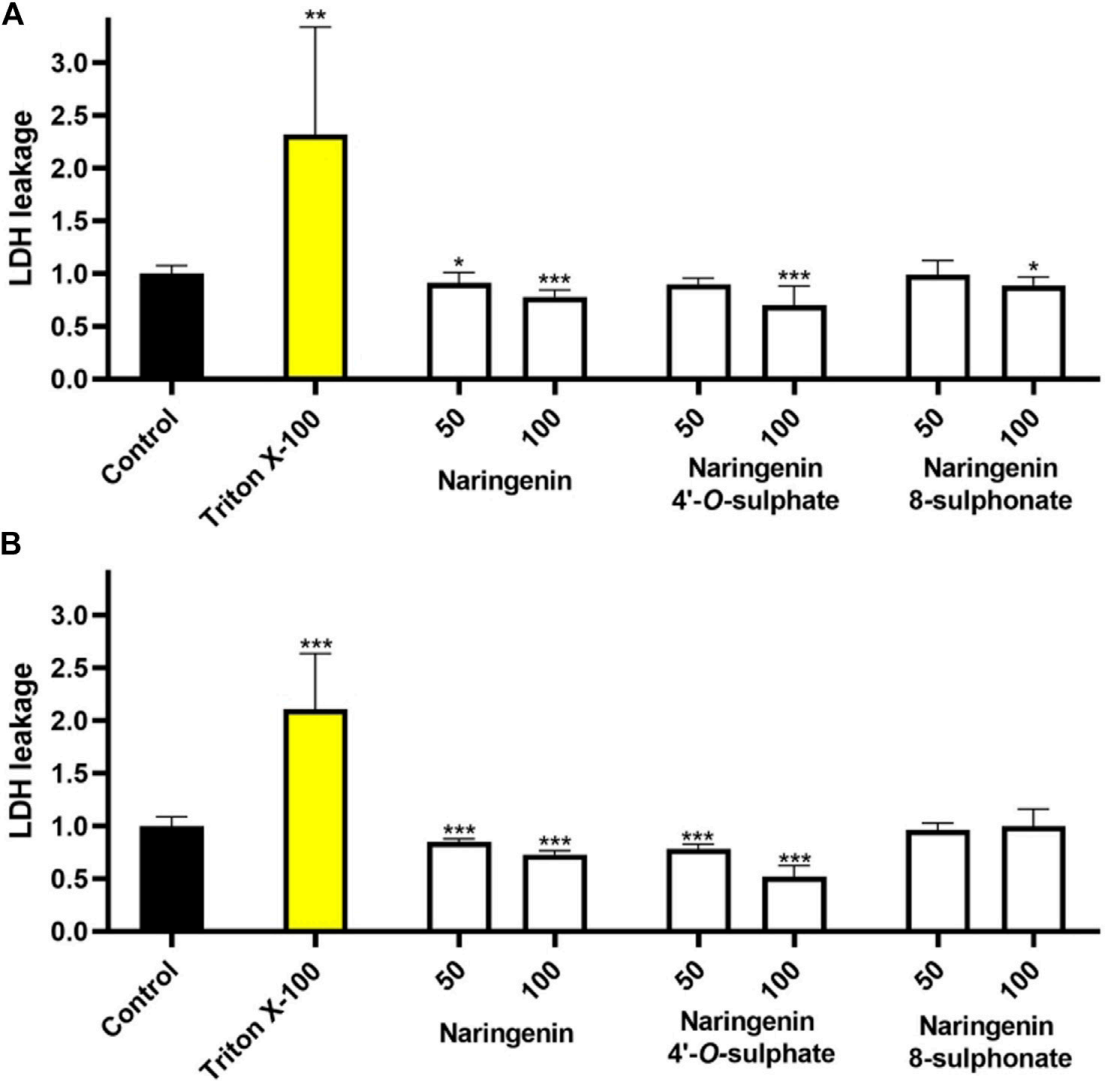
Effects of naringenin, naringenin 4’-*O*-sulphate and naringenin 8-sulphonate, at concentrations of 50 and 100 μM, on the membrane integrity of HaCaT **(A)** and MRC-5 **(B)** cells. Triton X-100 (1%) was used as positive control. Results are expressed as mean of, at least, three independent experiments, each performed in triplicate. ***p < 0.001.

The assessment of each molecule upon membrane integrity revealed that none of the compounds increased the extracellular LDH levels in any of the cell models (Figure 4). These results, which point to the absence of necrosis, together with the minimal impact of the compounds on cell viability, suggest that the compounds are not cytotoxic when used at the tested concentrations.

### 3.5 Genotoxicity

**FIGURE 5:**
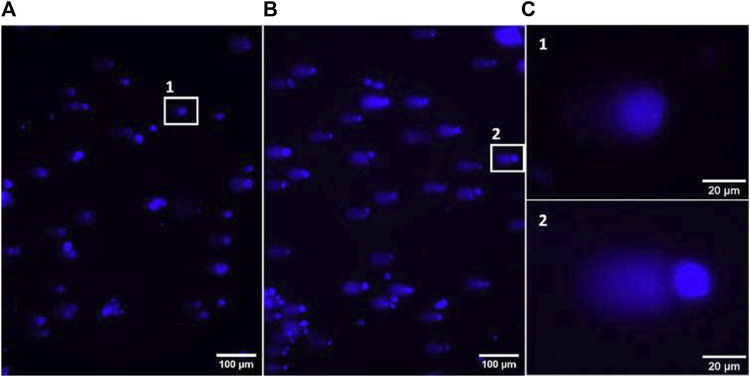
Representative images of fluorescence microscopy, using a ×10 objective, from the negative control **(A)** and positive MMS control **(B)** of the alkaline comet assay. Zoom in of representative comets from the negative and positive controls **(C)**.

Comet assay was performed to evaluate possible genotoxicity of compounds. This assay is the most widely used method to assess DNA damage in eukaryotic cells and is commonly used in human biomonitoring and clinical studies (Langie et al., 2015; Subash, 2016; Fatima and Yadam, 2023). It is based on the principle that, under electrophoresis, fragmented DNA migrates faster than intact DNA (Cordelli et al., 2021). As a result, an image evocative of a comet, where the DNA that migrates faster forms the comet tail, can be seen after analysis through fluorescence microscopy (Figure 5). The percentage of DNA in the tail may be used as a parameter to evaluate the damage on DNA (Clementi et al., 2021). In this work, compounds were tested at 100 μM, results were collected from three independent experiments, and a total of at least 220 comets were analysed for each of the conditions.

**FIGURE 6:**
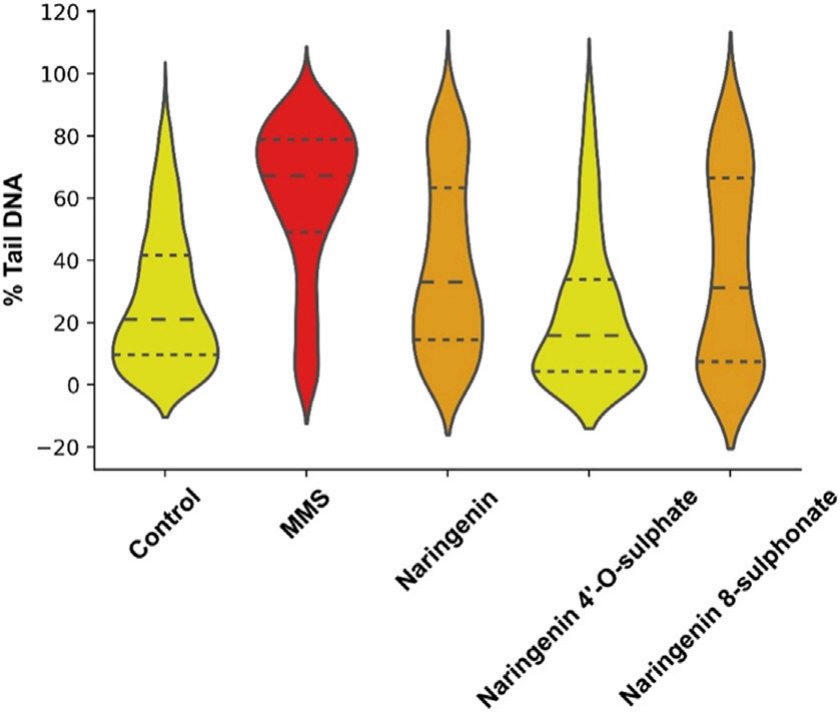
Violin plot representation of the distribution of DNA in tail percentage of each analysed cell for controls and treatments. MMS at 500 μM was used as positive control, - - - - = median; ------ = first and third quartiles.

Comparison of data distribution on the violin plot allows to see that there is a notorious difference between negative control and MMS positive control, which depicts the robustness of the method in detecting DNA damage. While in the negative control most of the cells have more DNA in the head of the comet, with MMS treatment most of them have more DNA in the tail. The treatment generating the most similar data compared to the negative control was with naringenin 4’-*O*-sulphate (Figure 6). Data distribution of results from naringenin and naringenin 8-sulphonate, despite being very distant from the positive control, seems to indicate that these treatments may produce some degree of genotoxic effects. In terms of statistics, the median of negative control was 20.98% of DNA in the tail, while for the positive control it was 67.12%. Regarding treatments, medians of 33.07%, 15.89% and 31.18% were obtained for naringenin, naringenin 4’-*O*-sulphate and naringenin 8-sulphonate, respectively (Table 1).

**TABLE 1:** First quartile, median and third quartile values for the distribution of cells regarding their percentage of DNA in tail.

Treatment	First quatile	Median	Third quartile
Control	9.62	20.98	41.62
MMS (500 μM)	48.99	67.12	78.85
Naringenin (100 μM)	14.24	33.07	63.36
Naringenin 4’-O-sulphate (100 μM)	4.33	15.89	33.90
Naringenin 8-sulphonate (100 μM)	7.25	31.18	66.61

Reports concerning the mutagenic effects of naringenin are not consensual. An alkaline comet assay performed to evaluate the mutagenic effects of naringenin in HT29 cells revealed no impact on DNA integrity using concentrations up to 100 μM (Baranowska et al., 2021). However, it may be important to refer that, despite the described methodology of this work being very similar to ours, there is no mention to a positive control, a critical part of methodological quality control, and none of the compounds caused more than 5% of DNA in comet tail, which is significantly lower than our negative control. These numerical differences may difficult the comparison of studies. In opposition, other alkaline comet assay that evaluated the effects of naringenin in different types of cells showed that naringenin was able to cause DNA damage in different cell lines, namely, MCF-7, HT-29, PC-12, and L-929 (Kocyigit et al., 2016). However, an effect in MCF-7 and HT-29 cells was only observed with concentrations up to 100 μM, pointing to the dependence of this biological effect on the cell lines used. A comet assay performed in human umbilical vein endothelial cells (HUVECs) also confirmed the ability of naringenin to cause DNA damage at concentrations from 50 μM (Çeker et al., 2019). Interestingly, the same study proved that naringenin could have antigenotoxic effects at lower concentrations, 8 μM causing the best effects (Çeker et al., 2019). This possibility of distinct effects depending on the concentration used is not considered by prediction models like ADMETlab, which take into account solely the chemical structure at face value, which may be seen as a disadvantage and reinforces the importance of experimental confirmation.

To the best of our knowledge, there are no reports on the effects of naringenin 8-sulphonate and naringenin 4’-*O*-sulphate on DNA, ours being the first. Regarding naringenin 4’-*O*-sulphate, these experimental results seem to agree with the ADMETlab 2.0 prediction, since the calculated mutagenicity probability was low. The same can be postulated for naringenin, considering that the mutagenicity probability was medium. However, naringenin 8-sulphonate prediction indicated values closer to naringenin 4’-*O*-sulphate, while the experimental values showed that it was closer to naringenin.

### 3.6 Permeability assay

**FIGURE 7:**
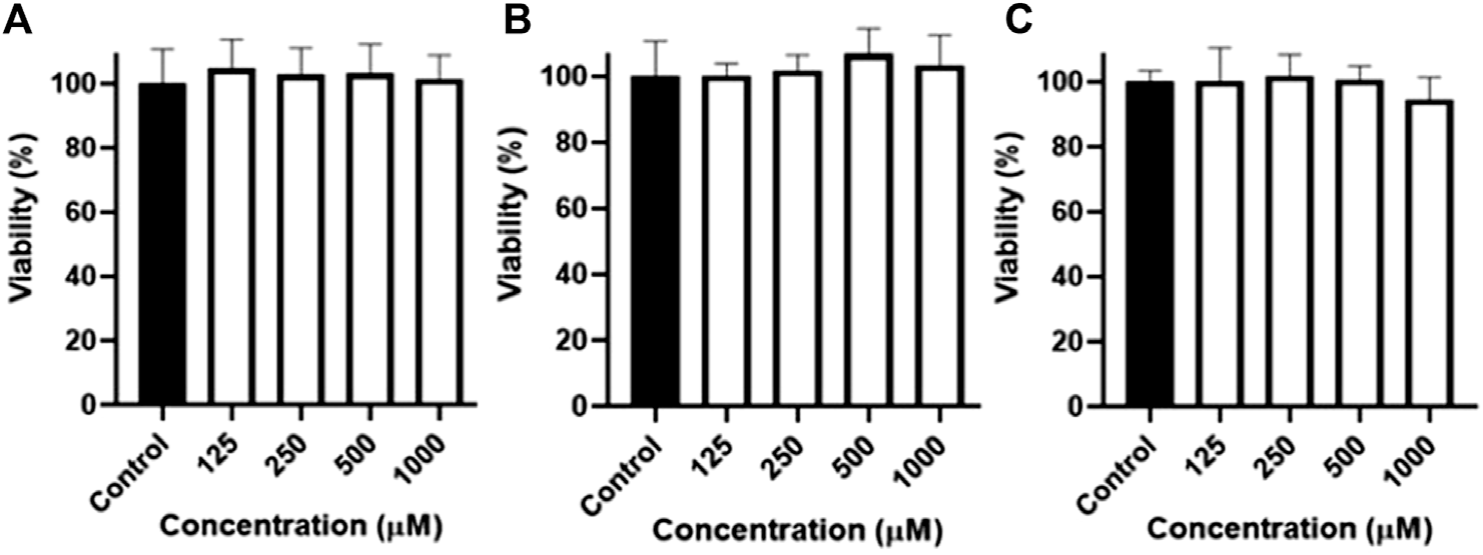
Viability of Caco-2 cells after 4 h of exposure to naringenin **(A)**, naringenin 4’-*O*-sulphate **(B)** and naringenin 8-sulphonate **(C)**, at concentrations ranging from 125 to 1,000 μM. Results are expressed as mean of, at least, three independent experiments, each performed in triplicate.

The ability of naringenin 8-sulphonate to cross biological barriers was tested using a Caco-2 permeability model. The first step for this assay was to test the possible impact of high concentrations of samples in Caco-2 cells during the time of the experiment. Thus, an MTT assay, using concentrations of naringenin, naringenin 4’-*O*-sulphate and naringenin 8-sulphonate until 1,000 μM, was performed. Since no significant changes on cell viability were detected at the highest concentrations (Figure 7), 1,000 μM was the concentration selected for the assay.

**FIGURE 8:**
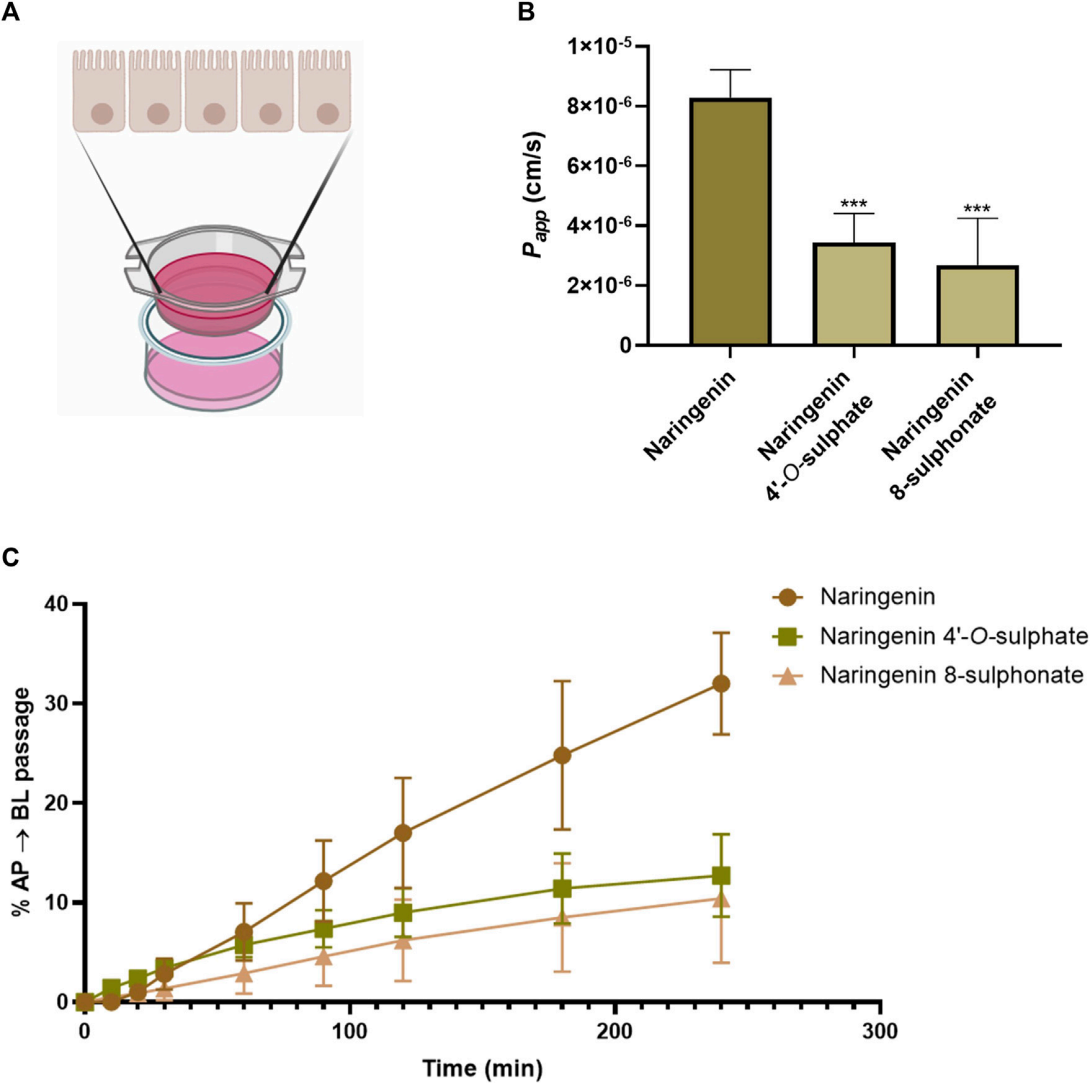
Schematic representation of Caco-2 permeabilty model. Drawn using elements from BioRender **(A)**. Cumulative percentage, as a function of time, of the initial amount of naringenin, naringenin 4’-*O*-sulphate and naringenin 8-sulphonate that was detected in the basolateral compartment after crossing the Caco-2 cell monolayer **(B)**. Apparent permeability coefficient (*P*_*app*_) values for naringenin, naringenin 4’-*O*-sulphate and naringenin 8-sulphonate across Caco-2 cell monolayers. ***p < 0.001 compared to naringenin **(C)**. Results are expressed as mean and were collected from three independent experiments, performed in duplicate.

Quantification of compounds in the basolateral chamber (Figure 8A) at each timepoint allowed to calculate their passage from the apical chamber along time. As can be seen in Figure 8C, few conclusions could be taken from the first 60 min of the assay, since the results are very similar, none of the compounds reaching 10% of passage. On the other hand, from 90 min onwards, a progressive highlight of naringenin is observed in comparison with its sulphur-containing derivatives. In fact, 32.01% of the amount of compound placed in the apical chamber in the beginning of experiment was found in the basolateral chamber after 240 min, while 12.73% and 10.41% were the maximum percentages obtained for naringenin 4’-*O*-sulphate and naringenin 8-sulphonate, respectively. This last time point was used to calculate the *P*_*app*_ coefficient, a parameter used for the classification of drugs permeability. Thus, according to the literature, the compound permeability is considered as low when *P*_*app*_ is inferior to 1 × 10^−6^ cm s^-1^, moderate when it is between 1 × 10^−6^ cm s^-1^ and 1 × 10^−5^ cm s^-1^ and high when it is superior to 1 × 10^−5^ cm s^-1^ (Karakucuk et al., 2021). In this work, naringenin 8-sulphonate exhibited a *P*_*app*_ of 2.68 × 10^−6^ cm s^-1^, while the values for naringenin 4’-*O*-sulphate and naringenin were 3.45 × 10^−6^ cm s^-1^ and 8.29 × 10^−6^ cm s^-1^, respectively (Figure 8B). Converting the results from the ADMETlab 2.0 to linear values, we obtained 3.70 × 10^−6^ cm s^-1^, 3.71 × 10^−6^ cm s^-1^ and 1,57 × 10^−5^ cm s^-1^ for naringenin 8-sulphonate, naringenin 4’-*O*-sulphate and naringenin, respectively (Table 2). While both experimental and predictive values placed the two first compounds as moderate in terms of permeability, naringenin permeability fits in the moderate category following our experimental results, but it is considered high when we look at the ADMETlab 2.0 prediction.

**TABLE 2:** *P*_*app*_ (cm s^-1^) values predicted with ADMETlab 2.0 and experimentally determined with Caco-2 permeability model.

Compound	ADMETlab 2.0	Experimental
Naringenin	1.57 × 10^−5^ ↗	8.29 × 10^−6^ →
Naringenin 4′-*O*-sulphate	3.71 × 10^−6^ →	3.45 × 10^−6^ →
Naringenin 8-sulphonate	3.70 × 10^−6^ →	2.68 × 10^−6^ →

→, moderate *P*_*app*_; ↗, high *P*_*app*_ (Karakucuk et al., 2021).

Through the calculation of the final mass balance, by comparing the amount initially applied with the sum of the amount collected from the basolateral side along the experiment and the amount that remained in the apical side, it was possible to conclude that there were no losses of compounds during the assay.

It is described in literature that flavanones show higher permeability across Caco-2 monolayers when compared to other flavonoid classes (Fang et al., 2017). It is also demonstrated that this permeability may be markedly decreased when they are glycosylated, since it reduces lipophilicity (Fang et al., 2017; Najmanova et al., 2020), which is in line with several studies that have shown that the decrease of lipophilicity is correlated with lower permeability (Arnott and Planey, 2012; Najmanova et al., 2020). As in the case of flavonoid glycosides, flavonoid sulphates and sulphonates are also less lipophilic than the respective aglycones, which hinders their permeability (Arnott and Planey, 2012). Besides, these molecules have a higher MW, which is another factor against the capacity to be absorbed (Arnott and Planey, 2012). Regarding the compounds of this work, as far as we know, there are no previous reports about naringenin 4’-*O*-sulphate and naringenin 8-sulphonate concerning this matter. In opposition, naringenin permeability was already investigated in Caco-2 cells, with reported *P*_*app*_ values between 1.26 × 10^−5^ cm s^-1^ and 5.28 × 10^−5^ cm s^-1^ (Nait et al., 2009; Tian et al., 2009; Kobayashi et al., 2012). While the predictive value from ADMETlab was comprised in this range, our experimental results indicated a lower value. Even so, it is important to note that the reported values were obtained using different compound concentrations and incubation times. The ability of naringenin to cross Caco-2 has been associated with passive diffusion and active transport mechanisms by different studies (Nait et al., 2009; Kobayashi et al., 2012), one of them suggesting that the active transport is mediated by the MRP1, which is expressed in the basolateral side of the intestinal barrier (Nait et al., 2009). Since there are no reports regarding the transport of drug sulphate conjugates by MRP1, neither about the inhibition of this protein by those metabolites (Jarvinen et al., 2021), the impact of sulphation on the naringenin active transport may be difficult to predict.

## 4 Conclusion

Results from previous work indicated that naringenin 8-sulphonate is more active than naringenin and naringenin 4’-*O*-sulphate in terms of anti-inflammatory activity. However, the results presented herein point to less ideal ADMET properties. In fact, naringenin 8-sulphonate was the only compound from this study that did not fulfil all the ADMETlab 2.0 *in silico* tool criteria for proper drug-likeness and the one with the worst QED predictive value. Experimentally, it did not reveal significant cytotoxicity, but it exhibited some genotoxicity in the alkaline comet assay, as well as naringenin, and unlike naringenin 4’-*O*-sulphate. This data indicates that the presence of the sulphur containing group at the position 4’ seems to nullify the genotoxic effects of naringenin, while its presence at the position 8 did not, highlighting the importance of the group position for the toxicological properties of this polyphenol. To understand whether this behaviour can be extrapolated for other flavonoids or is specific for this molecule, it would be interesting to study the impact of 4′ substitution on the mutagenicity of other flavonoids. Regarding permeability, as expected from the predicted values, the presence of a sulphur containing group greatly reduces the permeability of the molecule. Even so, naringenin 4’-*O*-sulphate and naringenin 8-sulphonate permeability may be considerate as moderate, reaching values even higher than some flavonoid aglycones, such as myricetin and morin, which have reported *P*_*app*_ values of 1.70 × 10^−6^ cm s^-1^ and 0.62 × 10^−6^ cm s^-1^, respectively (Tian et al., 2009). In summary, the better pharmacological effects of naringenin 8-sulphonate in comparison with naringenin 4’-*O*-sulphate and naringenin, previously reported by our research group, may be, somehow, counterbalanced by its less favourable ADMET properties.

## Data Availability

The original contributions presented in the study are included in the article/Supplementary Material, further inquiries can be directed to the corresponding author.
